# A Case for Mental and Physical Rest in Youth Sports Concussion: It’s Never too Late

**DOI:** 10.3389/fneur.2012.00171

**Published:** 2012-12-11

**Authors:** Rosemarie Scolaro Moser, Philip Schatz

**Affiliations:** ^1^Sports Concussion Center of New JerseyLawrenceville, NJ, USA; ^2^International Brain Research FoundationFlanders, NJ, USA; ^3^St. Joseph’s UniversityPhiladelphia, PA, USA

**Keywords:** cognitive rest, concussion, mild traumatic brain injury, sports concussion, treatment of concussion, youth concussion

## Abstract

Over the past decade, there has been a considerable increase in research on, and media attention to, sports-related concussion. However, despite accurate diagnosis, effective treatment and management of sports-related concussion have remained a challenge. There are approximately 1.8 million traumatic brain injuries in the United States annually (Faul et al., 2010) and emergency department pediatric visits for suspected concussion have doubled in the past decade (Bakhos et al., 2010). However, health care providers and medical researchers have yet to offer an effective, reliable evidence-based treatment for concussive brain injury. The Zurich 2008 Consensus Statement on Concussion in Sport codified the prescription for cognitive and physical rest immediately following a concussion based on clinical acumen and common sense (McCrory et al., 2009). Currently, rest is the considered the best *immediate* treatment for concussion. Other supportive and anecdotal treatments are often applied throughout the post-concussive recovery process to address persistent symptoms. The need for empirical research to translate current guidelines for rest into evidence-based treatment protocols is essential. A recent study evaluated the efficacy of comprehensive rest and concluded that such rest may be helpful whether applied soon after a concussion or weeks to months later (Moser et al., 2012). Here, we present a case illustrating the effectiveness of rest in a youth athlete, commenced after experiencing 13 months of post-concussion symptoms. There appears to be value in applying a specific period of cognitive and physical rest following concussion, whether immediately or later in the recovery phase.

Sports concussion has become a public health concern, especially for youth participating in sports activities (Moser, 2007). From 1997 to 2007, visits to emergency departments for sports concussion doubled in the 8–13 year old group; in 14–19 year olds, the incidence had increased by more than 200% (Bakhos et al., 2010). National organizations have established position statements regarding the identification and management of concussion (AAN, 1997, 2010; Guskiewicz et al., 2004; Moser et al., 2007). The Centers of Disease Control and Prevention have introduced educational programming and materials for schools, physicians, teams, and the general public in an effort to aggressively address this problem (CDC, 2010). Furthermore, states in the USA are adopting legislation and passing laws that require schools and organized athletics to create and maintain concussion management policies and programs that reflect the most up-to-date guidelines. As of this writing, over 40 states have already passed some type of sports concussion (Barton, 2011; Moser, 2012).

Since the most recent 2008 Zurich International Conference on Concussion in Sport (McCrory et al., 2009), health care professionals have experienced increased pressure to become educated and adopt the most up-to-date guidelines for concussion identification, treatment, and management. The AAN guidelines for concussion diagnosis and management (AAN, 1997) are now antiquated, although there are still health care practitioners who continue to employ the old grading system. AAN is working to update these guidelines in 2012. Nonetheless, quickly evolving guidelines, the proliferation of research, as well as high profile medical malpractice lawsuits (Schwarz, 2010), are challenging pediatricians, primary care physicians, and emergency department physicians to rapidly increase the slopes of their learning curve.

A concussion is a mild traumatic brain injury (TBI), defined by any alteration in consciousness due to a blow or strong force to the head. The pathophysiology of concussion has been well documented as a neurometabolic cascade that results in altered cerebral blood flow and glucose metabolism preceded by axonal depolarization and ionic changes (Giza and Hovda, 2001). For 80% of youth athletes, concussion symptoms usually resolve within 3 weeks (Collins et al., 2006), and it is widely accepted that youth athletes tend to take longer to recover and tend to be more symptomatic than adults (McCrory et al., 2009). Youth athletes are also more susceptible to the condition of Second Impact Syndrome, a neurologically serious condition that can result in death when a concussed youth receives another hit or force to the head prior to recovery from the first event (Cantu, 1998). Physiologically, this syndrome has been demonstrated in animal models (Vagnozzi et al., 2007, 2008). The Zurich guidelines (McCrory et al., 2009) outline the importance of proper identification of concussion, the need to remove the athlete from play, and to only return the athlete when asymptomatic, after proper neurocognitive assessment, physical exertional assessment, and a graded program of exertion prior to medical clearance. Since symptoms may not fully manifest until days after the event, no youth suspected of having sustained a concussion should ever return to play the same day under any conditions (McCrory et al., 2009).

The recent findings from the Center for the Study of Chronic Traumatic Encephalopathy (CTE) are sobering (McKee et al., 2009). Having confirmed CTE in select NFL football players in their 40s, McKee and colleagues (2009) have now identified tau proteins, the neuropathologic “marker” of CTE, in an 18 year old high school football player and in a collegiate freshman football player (Stern et al., 2011). Thus, the implications for caution in the management of concussion in youth have never been more pressing: the importance of proper identification and treatment is obvious.

Although self-report of symptoms remains the most common method of identification, use of neurocognitive testing (baseline and post-concussive assessment of memory, attention, reaction time, and mental speed) and use of balance and physical exertional testing are of significant value in diagnostic clinical decision-making (Fox et al., 2008; Covassin et al., 2012). Furthermore, numerous experimental research methodologies are being applied in an effort to better identify and understand concussion. For example, neuroimaging studies using functional MRI have captured the cognitive “activation patterns” of concussed athletes (Chen et al., 2004). Electrophysiological assessment (such as EEG) has been used to document abnormalities in neurophysiology of brain function (Gosselin et al., 2006). Other technologies, including Diffuse Tensor Imaging (DTI), Susceptibility-Weighted Imaging (SWI), Photon Emission Tomography (PET), and Magnetic Resonance Spectroscopy (MRS), are garnering experimental interest (Gardner et al., 2012; Maugans et al., 2012). In addition, the role of blood biomarkers in TBI has been hailed as a recent breakthrough in military research, although its application to concussion is not apparent at this time (Zaroya, 2010). While strides are being made in diagnosis and identification, there appears to be limited research on treatment of concussion. Although there are a number of traditional supportive, alternative, and anecdotal treatments, there is little if any randomized, scientifically controlled evidence upon which to base these treatments.

## Treatment and Management of Concussion

For the most part, concussions typically resolve within a week or more in most cases, and for youth are usually fully resolved within 3 weeks (Collins et al., 2006). Thus, cognitive and physical rest, especially immediately following a concussive injury, has been promoted as the most effective treatment for concussion (McCrory et al., 2009). This term is loosely defined, but can be operationally defined as: (1) time off from school, (2) no homework, (3) no reading, (4) no visually stimulating activities, such as computers, video games, texting, use of cell phones, and limited or no TV, (5) no exercise, athletics, chores that result in perspiration/exertion, (6) no trips, social visits in or out of the home, and (7) increased rest and sleep.

The case for rest has some support in translational animal model research. It has been demonstrated that voluntary exercise in concussed rats may delay recovery if administered to soon following the TBI (Griesbach et al., 2004). Similarly, cerebral hyperactivation on fMRI scans of high school athletes during the first week post-concussion predicted a prolonged clinical recovery compared to high school athletes who did not evidence hyperactivation (Lovell et al., 2007). Findings from this latter study suggest that activating the brain immediately following a concussion may result in a longer period of post-concussion symptoms.

For protracted cases in which concussion symptoms last more than a month, pharmacologic treatments may be employed to address the concussion symptoms of headache, sleep disturbance, and changes in emotional affect (Lucas, 2011; Petraglia et al., 2012). In these cases, a comprehensive neurological evaluation is also helpful to rule out other co-existing conditions that may complicate the concussion picture, such as anemia, hypoglycemia, seizure disorder, migraine history, allergies, or more serious brain pathology. More recently, in select cases of mild TBI with prolonged recovery, Amantadine has been employed. In a small, limited study of pediatric TBI, Amantadine was thought to have positive effects as measured by behavioral and neuropsychological testing (Beers et al., 2005).

Aside from pharmacologic interventions, biofeedback has been utilized to address headache, and psychotherapy to address emotional and behavioral adjustment (Andrasik, 2010). Neurofeedback, although still considered experimental, is employed to “normalize” brain wave patterns, in the hopes of reducing symptoms, although rigorously controlled research has not yet clearly demonstrated its efficacy (Thatcher, 2009; Thornton and Carmody, 2009). Vestibular therapy has shown promise for post-concussive symptoms of dizziness and balance problems (Alsalaheen et al., 2010). In addition, examples of other anecdotal, alternative/complementary treatments include Epsom salts footbaths (for magnesium intake), craniosacral therapy, and nutraceuticals such as Omega 3 fatty acids (Greenman and McPartland, 1995; Lewis and Bailes, 2011; Moser, 2012).

Thus, for the most part, treatments for concussion have not been aggressively researched or validated. In fact, the most typical and recommended treatment, that of cognitive and physical rest, up until recently, was not evidence-based. However, Moser et al. (2012), in a study of 49 high school and collegiate athletes, documented that a period of cognitive and physical rest, whether applied immediately or in a delayed fashion, may be efficacious in treating concussion. Findings demonstrated improvements on neuropsychological testing and in symptoms after a period of 1 week of rest with significant comparable improvement despite time from concussion (1–7 days vs. 8–30 days vs. 31+ days). This research was not without its limitations as it this was a convenience sample, without a control group and the study was not randomized and blinded. Furthermore, factors such as the quality of rest, specific demographic variables, were not studied in detail.

## Case Presentation

The implementation of cognitive and physical rest immediately following a concussion has been typically recommended as the “cornerstone” of concussion treatment (McCrory et al., 2009; Moser et al., 2012). The following is an illustrative case study of one of the participants of the recent rest study noted above (Moser et al., 2012). In this case, rest had not been fully implemented until 13 months of post-concussion syndrome. Importantly, however, the patient had sustained a total of four concussions over this period of time, the last of which had been approximately 3 months prior to her examination described below.

Lindsay (not her actual name) was a 14 year old basketball player who arrived for a neuropsychological consultation with presenting complaints of “memory loss, anxiety, irritability, reading difficulty,” mental slowness, fatigue, decline in school grades, and a recent school suspension for a prank. Prior to her injuries she had been a “straight A student…in a special school leadership program.” She had a previous history of four concussions, prior to her first visit for neuropsychological consultation at the Sports Concussion Center of New Jersey.

Concussion 1: 13 months prior to exam, Lindsay’s head hit the floor during a basketball game. There was no loss of consciousness (LOC), but possible amnesia was described. Symptoms of headache, balance problems, nausea, and confusion were reported with symptoms that persisted “for weeks.” She was examined by her primary care physician and advised to avoid gym for 4 days and basketball for 2 weeks. There were no other cognitive or physical restrictions, but her symptoms lingered.
Concussion 2: Approximately 2 weeks later, Lindsay sustained an elbow to the head during a basketball game. Again, no LOC was reported, although there was possible amnesia. Her headaches continued along with memory problems, and Lindsay was kept out of sports for 2 weeks with no other restrictions. Despite this, symptoms did not completely resolve.
Concussion 3: Approximately 2 weeks later, Lindsay fell in gym class and hit her head to floor. This time there was a brief LOC. Her mother indicated that Lindsay’s anxiety “came on like a storm…almost impossible to speak to her logically…” Thus, Lindsay was referred for psychiatric treatment and started on a regimen of Zoloft.
Concussion 4: Approximately 10 months later, Lindsay hit her head on the floor during a basketball game, with no LOC reported. However, she continued to experience post-concussion symptoms that had not previously resolved.

Prior to the neuropsychological consultation, Lindsay had been followed by her pediatrician, had had multiple visits to the hospital Emergency Department, and had undergone MRI that was interpreted with negative findings. Lindsay had never taken any time off from school and was never provided any academic accommodations. There was no reported history of significant medical difficulties/disorders/diseases, early childhood or developmental difficulties, mental health difficulties, alcohol/substance use issues, attention deficit disorder, learning disorder, or behavioral problems.

Her parents were not knowledgeable about sports concussion and current identification and management guidelines for youth. They were led to believe by her health care providers and the school that her recent cognitive, emotional, and behavioral difficulties (which had not existed premorbidly) were due to a psychological condition and she was provided psychiatric treatment and prescribed Zoloft.

At the time of the initial neuropsychological consultation at our Center, the Immediate Post-Concussion Assessment and Cognitive Testing (ImPACT), a computerized neuropsychological test battery, was performed (Maroon et al., 2000). Although there was no pre-concussion baseline for comparison, Lindsay’s scores were compared to norms for female athletes in her age group. Her scores at the time of the initial exam were quite low (see Table 1). Verbal Memory was below the first percentile and Visual Memory was at the first percentile. Visual Motor Speed and Reaction Time percentiles were also below average. Impulse Control score was within the normal range, providing evidence for a valid test administration (Lovell, 2004; Schatz et al., 2012). The Cognitive Efficiency score, which represents the athlete’s ability to maintain a balance between speed and accuracy in responding to the test items, was low (below 0.20 is a questionable score whereas 0.34 is considered a mean score).

**Table 1:** **Case study ImPACT results (no baseline performed)**.

Composite	Post 1	2 week rest	Post 2	Post 3	Post 4	Post 5
Verbal memory	62 (<1%)		96 (85%)	97 (88%)	98 (88%)	100 (97%)
Visual memory	43 (1%)		79 (63%)	70 (43%)	79 (63%)	97 (99%)
Vis. motor speed	32.4 (17%)		35.4 (34%)	42.7 (80%)	36.2 (37%)	35.8 (35%)
Reaction time	0.68 (16%)		0.61 (38%)	0.58 (53%)	0.60 (42%)	0.56 (64%)
Impulse control	6		8	6	6	4
Total symptoms	36		4	2	1	1
Cog. efficiency	0.26		0.51	0.49	0.45	0.52

Because of the intensity of her symptoms, the length of her post-concussion syndrome, and the number of previous concussions, she was advised to take a medical leave from school for a period of 2 weeks with a regimen of cognitive and physical rest (as described above). In addition, a consultation with a neurologist was recommended. Lindsay and her parents were skeptical of what they perceived as such a drastic recommendation for a medical leave from school and academics. They were then educated about the need for the brain to recover from the neurometabolic cascade that takes place in response to concussion (decreased cerebral blood flow, dysregulation of ions and neurotransmitters) and that this is accomplished with strict rest, instead of continued brain activation that might further deplete available cerebral energy stores. They were explained that the neurometabolic cascade typically occurs early in the post-concussion stage and that given her complex case, there was no guarantee for her recovery, but that perhaps rest could still help her.

Lindsay returned for a second consultation after 2 weeks of compliant rest. By that time, her ImPACT scores were now remarkably better, and well within in the normal range (see Table 1). At that time, Lindsay stated that she felt “85–90%.” Her Symptom Index had decreased from 36 to the normal range of 4. Both she and her parents marveled at the effect of a strict regimen of cognitive and physical rest. The improvement in scores could not be attributed to possible invalidity or “faking bad” of test results during the initial consultation because examination of the test results revealed no indicators of invalidity (Schatz et al., 2012). Neurological consultation had in the meantime been conducted and confirmed a diagnosis of post-concussion syndrome and EEG testing was within normal limits. MRI was not repeated as it had been performed a few months prior to the consultation with negative findings. Pharmacologic treatment for headache, other than over the counter meds, was rejected by the patient.

In week three, Lindsay had returned to school on a part time basis. With academic accommodations, she was permitted to arrive at school later in the morning, but she was still not permitted to complete homework or tests or use computers. Gym and physical activity were still restricted, as well as after school activities, and she continued to maximize sleep and rest. By the end of week three, she had passed exertional testing conducted by a certified athletic trainer and began reconditioning in a graduated, stepwise program. By week six, she was attending full days of school and had progressed to completing regular school work. Gym was still prohibited but sport specific drills were allowed. By week eight, Lindsay felt, in her own words, “AWESOME.” Her ImPACT scores were relatively stable and strong. She was cleared to return to gym and athletics by consensus of her neurologist, the athletic trainer, and the neuropsychologist (RSM). At that time, athletic activities with a low risk of contact were then recommended. Lindsay continued on a less frequent basis with her psychiatrist, aiming toward termination.

**Figure 1:**
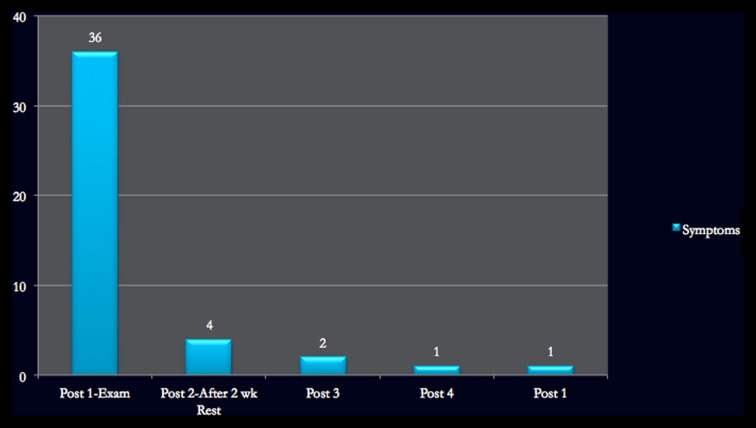
**Symptom checklist on ImPACT**.

**Figure 2:**
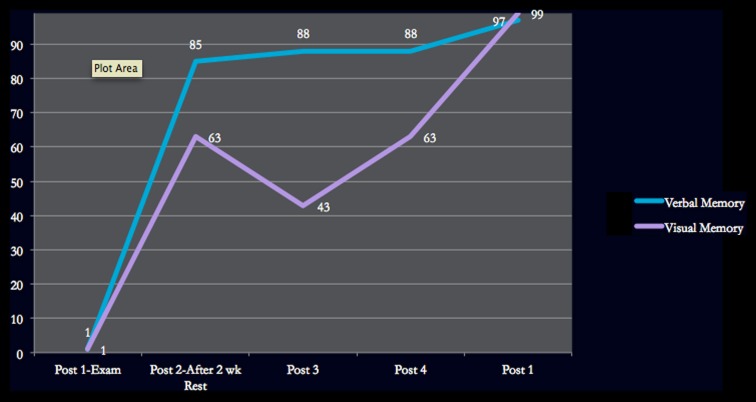
**Verbal and Visual Memory ImPACT scores**.

**Figure 3:**
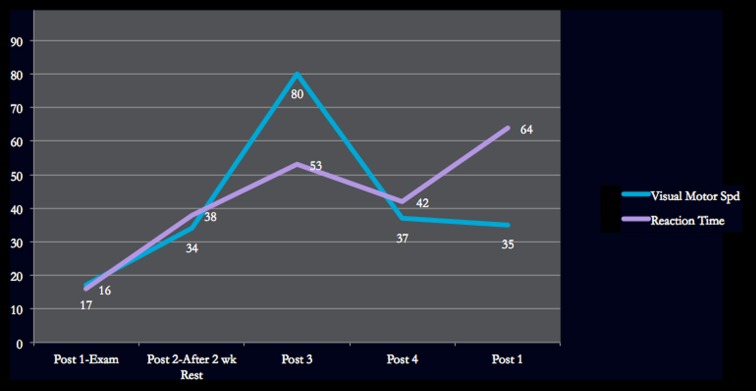
**Visual Motor Speed/Reaction Time ImPACT scores**.

**Figure 4:**
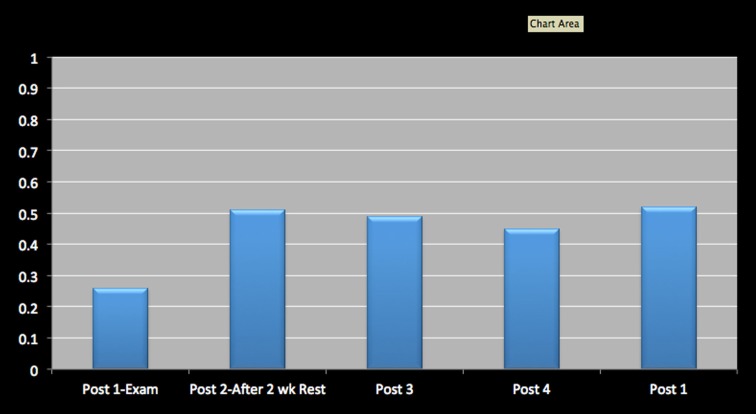
**Cognitive Efficiency on ImPACT**.

Figures 1–4 provide graphic representation of Lindsay’s improvement over the course of five consultation sessions held over the period of 8–9 weeks.

## Discussion

The above case may be exceptional in its resolution of a longstanding post-concussion syndrome with multiple concussions. The use of neurocognitive testing in this case, even without baseline test results for comparison, provided evidence of improvement and helped to guide clinical management. When test scores seemed stable and symptom scores were normal, the patient could then be progressed to physical exertional testing.

It could well be argued that her improvement was due to a placebo effect or mental health issues. However, Lindsay was just one of a number of patients who improved following delayed comprehensive rest that were included (de-identified and analyzed by independent analysis) in the Moser et al. (2012) study described above. In addition, her most recent concussion had only been approximately 3 months prior.

With the increased prevalence of sports concussion in youth, and the potentially enduring effects that have been correlated with repeated concussions (Moser et al., 2005; Schatz et al., 2011), it is imperative that research be guided toward finding effective empirically based treatments that will shorten recovery periods, reduce symptomatology, and prevent post-concussion syndrome. Such advances would not only be of benefit to youth, but would have greater applications across other injury populations, such as motor vehicle head traumas and possibly military blast injuries.

From these findings, it is plausible that a period of comprehensive rest may be beneficial even when applied 3 months after injury, or longer. To be clear, in defining comprehensive rest, we are recommending: no school/work attendance, no homework, no user of computers, no visually stimulating activities (texting, reading, FaceBook, videogames, drawing, playing a musical instrument), no physical activities (exercise, chore, activities that result in perspiration), no driving, no or very limited TV, no travel outside of the home, no family/social visits in or outside the home, and plenty of rest and sleep. Unfortunately, in the present authors’ experience, concussed individuals’ are often prescribed an undefined period of rest during which they unwittingly continue to perform academic or work duties at home while on “medical” leave.

Nevertheless, rest as a construct will need to be more accurately defined in order to inform research investigation. Factors such as *type* of rest (physical vs. cognitive), *length* of rest period, and *degree* or nature of rest (for example, no school vs. partial school days or school attendance but no note-taking, homework, tests, computers, etc.) should be considered in any research design. In addition, variables such as time since the concussion before rest is administered, severity of concussion symptoms at rest onset, and number of previous concussions in the patient’s history could very well determine the efficacy of rest as a treatment. Consideration of these kinds of variables, in addition to typically considered variables of age, gender, learning disorders/ADHD, intellectual ability, and years playing sports, will hopefully aid in more accurate predictions for the recovery period.

The case presented here as an example of the application of rest may be illustrative and instructional, however it is only an “N of 1” and, as such, prohibits generalization. Although rest was effective at such a delayed point in the concussion history, one cannot reliably expect rest to be as successful in all cases of prolonged post-concussion syndrome. We will need to identify what variables, if any, will modify the efficacy of rest. Neuroimaging and electrophysiological assessment tools may assist us in better understanding the mechanisms of rest and help identify the brain activation patterns associated with different kinds of rest. With so little currently known about rest from a research perspective, it is hoped that this case study will stir interest in this deceptively simple, yet really complex, construct and important treatment for concussion, even months after the impact.

## Conflict of Interest Statement

The authors declare that the research was conducted in the absence of any commercial or financial relationships that could be construed as a potential conflict of interest.
